# Draft Genome Sequence of *Advenella kashmirensis* Strain W13003, a Polycyclic Aromatic Hydrocarbon-Degrading Bacterium

**DOI:** 10.1128/genomeA.00003-14

**Published:** 2014-01-30

**Authors:** Xinxin Wang, Decai Jin, Lisha Zhou, Liang Wu, Wei An, Lin Zhao

**Affiliations:** a School of Environmental Science and Engineering, Tianjin University, Tianjin, China; b China Offshore Environmental Service Co. Ltd., Tianjin, China; c College of Resources and Environment, University of Chinese Academy of Sciences, Beijing, China; d Shenzhen Key Laboratory of Environmental Microbial Genomics and Application, BGI, Shenzhen, China; e College of Environmental Science and Engineering, Ocean University of China, Qingdao, China

## Abstract

*Advenella kashmirensis* strain W13003 is a polycyclic aromatic hydrocarbon (PAH)-degrading bacterium isolated from PAH-contaminated marine sediments. Here, we report the 4.8-Mb draft genome sequence of this strain, which will provide insights into the diversity of *A. kashmirensis* and the mechanism of PAH degradation in the marine environment.

## GENOME ANNOUNCEMENT

Polycyclic aromatic hydrocarbons (PAHs) are widely distributed in marine sediments. These hydrocarbons can be degraded by many bacteria, which have attracted increasing attention (1). *Advenella kashmirensis* W13003 was isolated in PAH-contaminated sediments from Bohai Bay in China, and it can degrade PAHs, including pyrene and phenanthrene. Genomic analysis provides an opportunity to understand microbial PAH degradation (2–4). However, only one strain, i.e., *A. kashmirensis* WT001, has been sequenced (5). Here, the draft genome sequence of PAH-degrading *A. kashmirensis* W13003 is presented.

Genomic DNA was extracted using a commercial DNA isolation kit and sequenced using an Illumina HiSeq 2000 platform (San Diego, CA) with a whole-genome shotgun (WGS) strategy. The sequencing produced 9,871,420 paired-end reads with an average insert size of 300 bp, yielding about 200-fold coverage. The filtered reads were assembled, scaffolded, gap filled, and validated using SOAP*denovo* version 2.04 (6), SSPACE version 2.0 (7), GapFiller version 1.10 (8), and BWA version 0.7.4 (9). The final assembly consisted of 15 contigs, with the largest length being 1,071,036 bp, which were assembled into 12 scaffolds, the largest of which is 2,399,045 bp. The genome sequence was annotated using the NCBI Prokaryotic Genome Automatic Annotation Pipeline (PGAAP) (http://www.ncbi.nlm.nih.gov/genomes/static/Pipeline.html).

The genome consists of 4.8 Mb with a G+C content of 55.1%. A total of 4,507 coding sequences (CDSs), 33 pseudogenes, 1 noncoding RNA (ncRNA), 38 tRNA genes, and 1 rRNA operon were identified. Of the CDSs, 88.3% were assigned to clusters of orthologous groups (COGs), with amino acid transport and metabolism being the most abundant class, and 50.2% were annotated into 1,688 KEGG orthologous groups by using KAAS (10), involving 193 metabolic pathways. Clustered regularly interspaced short palindromic repeat (CRISPR) elements were not found using CRISPRFinder (11). The plasmid partitioning gene, *parB*, was detected on contig 7, which suggests the presence of plasmids. Eighty-nine tandem repeats were detected, as revealed by Tandem Repeats Finder version 4.07 (12). Only one incomplete prophage region was detected using PHAST (13). The IS3 and IS21 families dominate the insertion sequence (IS) elements, as revealed by ISFinder (14). An average nucleotide identity (ANI) analysis (15) revealed that *A. kashmirensis* W13003 is phylogenetically related to *A. kashmirensis* WT001 (78.7%).

Seven genes were identified as being involved in hydrocarbon degradation, including 2 alkane 1-monooxygenase genes, 1 protocatechuate 3,4-dioxygenase gene, 1 ring-cleavage dioxygenase gene, 1 biphenyl 2,3-dioxygenase gene, and 2 terephthalate 1,2-dioxygenase genes. Moreover, 6 genes were identified as being involved in compatible solute synthesis and uptake, including 3 glycine/betaine ABC transport genes, 2 betaine-aldehyde dehydrogenase genes, and 1 ectoine synthase gene, which may enhance tolerance to salt stress in the ocean. An inorganic sulfur compound oxidation gene cluster was detected similar to that of *A. kashmirensis* WT001. Information about the genome sequence of *A. kashmirensis* W13003 will be helpful for understanding the diversity of *A. kashmirensis* and the mechanisms of PAH degradation in the marine environment.

### Nucleotide sequence accession number.

The draft genome sequence of *A. kashmirensis* W13003 has been deposited in GenBank under the accession no. AYXT00000000. The version described in this paper is the first version.
